# Fundamentals of Cubic Phase Synthesis in PbF_2_–EuF_3_ System

**DOI:** 10.3390/ma19010195

**Published:** 2026-01-05

**Authors:** Sofia Zykova, Kristina Runina, Mariya Mayakova, Maria Berezina, Olga Petrova, Roman Avetisov, Igor Avetissov

**Affiliations:** 1 Department of Chemistry and Technology of Crystals Mendeleev, University of Chemical Technology of Russia, Miusskaya sq. 9, Moscow 125047, Russiakristinarunina95@gmail.com (K.R.); mn.mayakova@gmail.com (M.M.); maria_anyrova93@mail.ru (M.B.); armoled@mail.ru (R.A.); 2 Prokhorov General Physics Institute of the Russian Academy of Sciences, Vavilova Str., 38, Moscow 119991, Russia

**Keywords:** europium fluoride, lead fluoride, solid solutions, co-precipitation, solid-phase synthesis

## Abstract

**Highlights:**

- A high-purity cubic phase could be synthesized in the 0–37 mol% EuF_3_ composition range in a quasi-binary PbF_2_-EuF_3_ system.
- The ordered rhombohedral R-phase exists in the concentration range from 37–39 to 43–44 mol% EuF_3_ in the quasi-binary PbF_2_-EuF_3_ system.
- Isothermal cross-sections of T-X-Y projection of ternary Pb-Eu-F diagram in the 50–550 °C temperature range are reconstructed.

**Abstract:**

Fluoride solid solutions exhibit exceptional optical and thermodynamic properties that make them valuable for advanced technological applications, and the PbF_2_-EuF_3_ system represents a particularly promising quasi-binary system for developing high-performance materials. However, the comprehensive understanding of the thermodynamic conditions governing phase equilibria and the precise boundaries of homogeneity regions in this system remains incomplete, limiting the rational design of single-phase materials with desired properties. Therefore, we conducted a comprehensive investigation of the thermodynamic conditions (temperature and composition) controlling the existence of cubic and rhombohedral phases within the homogeneity regions of the PbF_2_-EuF_3_ system. Solid solution samples were synthesized using both solid-phase synthesis and co-precipitation techniques from aqueous nitrate solutions. Phase equilibria were systematically investigated in two critical regions: the solvus line spanning 0–10 mol% EuF_3_ and the ordered rhombohedral R-phase region spanning 35–45 mol% EuF_3_. Structural characterization was performed at temperatures below the phase transition temperature in lead fluoride (365 °C) using X-ray phase analysis, optical probing, and Raman scattering. Our investigation successfully demonstrated the possibility of obtaining cubic preparations of high purity across the 0–37 mol% EuF_3_ composition range. Additionally, we precisely defined the region of existence of the ordered rhombohedral R-phase within the concentration range of 37–39 to 43–44 mol% EuF_3_. These findings provide essential thermodynamic data for the rational design of PbF_2_-EuF_3_ solid solutions and establish clear compositional boundaries for obtaining desired phase structures in this technologically important fluoride system.

## 1. Introduction

Lead fluoride crystals have favorable properties such as high density, a non-hygroscopic and relatively chemically inert nature, and a short radiation length, while the scintillation light output is too small to obtain a photo-absorption peak under gamma ray excitation. Thus, it could not be used in medical imaging applications [1]. Lead fluoride crystals are widely used for the detection of beta rays due to their relatively small back scattering. Lead fluoride activated by europium (PbF_2_:Eu) is a light scintillator that is used for the detection of charged particles and soft gamma rays up to several hundred keV [2].

Timing performance of PbF_2_ crystals of various lengths and surface conditions coupled to SiPMs was evaluated against a reference detector with an optimized test setup using high-frequency readout and novel time walk correction, with special attention to the inherent limitations of single-photon Cherenkov detection only [3,4]. These materials also find application as up-conversion phosphors [5,6,7,8].

Ce-doped PbF_2_ crystals did not show intense photo- and radio-luminescence, and Eu- and Ho-doped ones showed several peaks excited under UV and 5.5-MeV alpha ray excitation, respectively [5].

In general, fluoride compounds and solid solutions in PbF_2_-REF_3_ systems (RE = rare earth element) are of interest for research as luminescent [9,10,11,12,13] and laser materials [14,15,16,17], especially effective in the mid-infrared range and in up-conversion imaging [13], as well as ionic conductors [18]. These systems have a wide crystallization region of the cubic fluorite phase *Fm*$\bar{3}$*m* (over 20 mol% at 650–700 °C) [9,10], but the phase diagrams and the phase existence regions at low temperatures (below 650 °C) are insufficiently studied. The complexity of studying these phase diagrams lies in the fact that lead fluoride undergoes a phase transition at 335–360 °C. The high-temperature β-PbF_2_ polymorph crystallizes in a fluorite-like cubic structure (*Fm*$\bar{3}$*m*), whereas the low-temperature α-PbF_2_ phase adopts an orthorhombic (*Pnma*) structure [9]. There is no reliable information about the homogeneity limits of PbF_2_, given the need to conduct research with highly pure materials. Typically, the declared chemical purity of the PbF_2_ under study is no more than 99.9 wt%.

PbF_2_ β ⟶ α phase transition is “frozen” because of the metastable state of β-PbF_2_ at room temperature. It allows using this material as a fluoride ion conductor and an optical material. When heated, the phase transition occurs easily, whereas when β-PbF_2_ is cooled from high temperatures, the phase transition is kinetically hindered.

In general, for the systems MF_2_–REF_3_ (where M = Ba^2+^, Sr^2+^, Ca^2+^, and Pb^2+^; RE = rare earth element), the presence of a rhombohedral ordered phase (R-phase) with the ideal composition M_4_RE_3_F_17_, existing in a relatively narrow concentration range of about 40 mol% REF3 [10,19,20,21,22,23], is assumed. According to the data [21], this phase is close to the fluorite phase, which complicates the identification of “rhombohedral fluorite”.

The phase diagram of the PbF_2_-EuF_3_ system is insufficiently explored—the high-temperature range of 650–1300 °C is only predicted [9] based on diagrams of other rare earth elements—both F- and R-phases were identified at 650 °C [10], and analysis conducted at 50 and 500 °C did not establish the presence of the R-phase, as this region was examined with a large interval of 10 mol%. Meanwhile, highly concentrated environments with reduced symmetry, activated by Eu, are promising materials for laser generation in the red spectral range [24].

**Figure 1:**
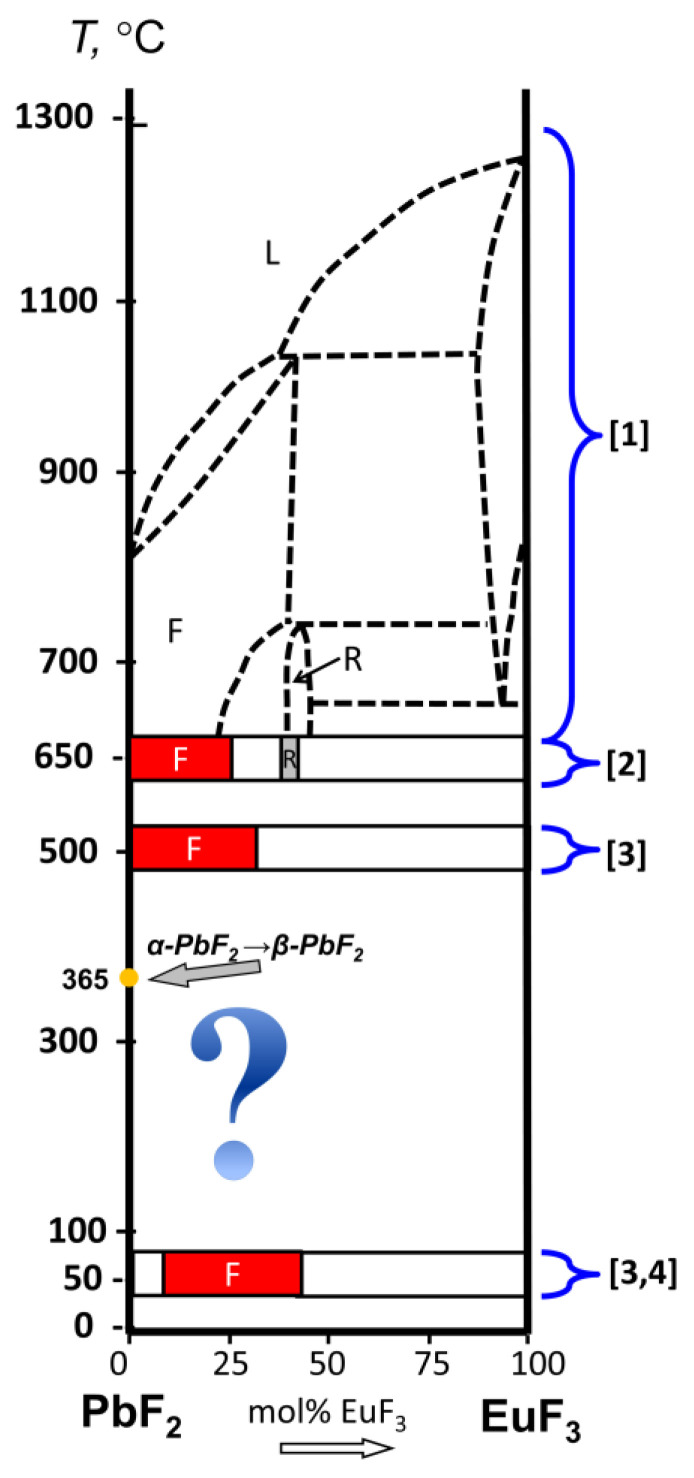
T-x diagram of PbF_2_-EuF_3_ quasi-binary system with the corresponding literature references for different temperature ranges: F—cubic phase; R—orthorhombic phase; L—liquid phase.

Studies of solid solutions of PbF_2_-EuF_3_ and glass–ceramic materials have shown that the introduction of EuF_3_ in concentrations of 7–10 mol% stabilizes the high-temperature phase even when synthesized at temperatures below 300 °C [11,12]. Thus, on the T-X diagram of PbF_2_-EuF_3_ at temperatures below 365 °C and EuF_3_ concentrations of 0–10 mol%, there should be a line between the regions of crystallization of a single cubic phase solid solution Pb_1−x_RE_x_F_2+x_ and the region of crystallization of two phases (solvus line). In the two-phase region, the above-mentioned solid solution phase and a phase extremely close to α-PbF_2_ are in equilibrium, as the solubility of RE in the low-temperature phase of lead fluoride is negligible (Figure 1).

It is known that impurities even in concentrations of hundredths of a mol% can significantly shift the boundaries of the homogeneity region, including the temperatures of polymorphic transitions [25].

The overall aim of this study was to clarify the boundaries of the homogeneity region of the cubic phase for future development of synthesis technology of single-phase extra-pure cubic phase preparations in PbF_2_-EuF_3_ system. To complete this task, we investigated two regions of the phase diagram of the quasi-binary system PbF_2_-EuF_3_ within the composition range of 0–45 mol% EuF_3_ and identified the R-phase within the composition range of 35–45 mol% EuF_3_. To date, low-temperature phase equilibria and the homogeneity range of the cubic phase and R-phase in PbF_2_–EuF_3_ system are still poorly constrained, which limits the rational design of luminescent materials based on this system.

## 2. Materials and Methods

Preparations within the PbF_2_-EuF_3_ system with EuF_3_ content ranging from 0 to 12 mol% and from 35 to 45 mol% were synthesized at a low temperature using the co-precipitation technique [12,26]. The starting materials included Pb(NO_3_)_2_ (99.99 wt%, LANHIT, Moscow, Russia), Eu(NO_3_)_3_ (99.99 wt%, LANHIT, Moscow, Russia), and HF (99.9 wt%, TECH Systems, Moscow, Russia) that were additionally purified to 99.99999 wt%. Initially, lead and europium nitrate solutions were prepared in bidistilled water (0.08 mol/L). Subsequently, these solutions were meticulously mixed in predetermined proportions. The resulting solution was added dropwise at 10 mL/min into the hydrofluoric acid solution (5 vol%) with continuous stirring, and a 10-fold excess of hydrofluoric acid was added to maintain a constant pH and T = 25 °C during the process. The process involved continuous stirring of the mother liquor using a magnetic stirrer. At this stage, a chemical reaction has been conducting:

(1 − *x*)Pb(NO_3_)_2_ + *x*Eu(NO_3_)_3_·6H_2_O + (2 + *x*)HF → Pb_1−x_Eu_x_F_2+x_↓ + H_2_O, (1)

The synthesis was carried out in a polypropylene reactor, and upon completion, the matrix solution was further stirred for one hour. The resulting precipitate was decanted, washed with bidistilled water until a negative reaction of diphenylamine to nitrate ions was observed, and then air-dried at 40–50 °C.

To determine the temperature of the phase transition in the concentration range of 0–9 mol%, EuF_3_ studies were performed on powder samples first obtained by co-precipitation at 50 °C and then subjected to heat treatment at certain temperatures of 200 °C, 300 °C, 330 °C, 350 °C, and 360 °C for 48 h in a resistance tube furnace in the first step. X-ray diffraction and luminescence spectroscopy were then analyzed. In the second step, the same samples were again heat-treated at the same temperature for an additional 24 h. A further set of studies was conducted. In all cases, the parameters remained unchanged. Therefore, we consider these data to be close to equilibrium. Because the transition from the high-temperature cubic phase to the low-temperature rhombic phase is “frozen,” quenching was not performed. The samples cooled from the treatment temperature to RT at an average rate of 50–100 °C/hour.

Preparations in the PbF_2_-EuF_3_ system with EuF_3_ concentrations ranging from 35 to 45 mol% were also obtained by solid-phase synthesis at 550 °C, which was higher than the reference α ⟶ β transition temperature. PbF_2_ (99.99% purity, LANHIT, Moscow, Russia) and EuF_3_ (99.99% purity, LANHIT, Moscow, Russia) were used as starting materials. Initially, the sampled powders were ground in a mortar to increase the contact area between the reacting substances. The solid-phase synthesis was conducted in a resistance handmade tube furnace at 550 °C for 2 h in corundum crucibles. Afterwards, the samples were cooled, and a series of studies were conducted. Then, the procedure of grinding was repeated, and the next heat treatment was performed under the same conditions for another 2 h. The same series of studies were repeated. Additionally, a fluorinating atmosphere was established [11,12,27,28] to prevent pyrohydrolysis. The results obtained after the first and second heat treatment were coincided.

The formation of a solid solution is described by the following chemical reaction.

(1 − *x*)PbF_2_ + xEuF_3_ → Pb_1−x_Eu_x_F_2+x_ (2)

After completion of the first stage, the resulting sintered powders were re-ground and placed in the furnace under identical conditions.

X-ray diffraction analysis was conducted using an INERL Equinox 2000 X-ray diffractometer (Inel SAS, Artenay, France) (CuK_α_ radiation with a wavelength of λ = 1.54 Å), with an accuracy of lattice parameter determination of +1%·and sensitivity up to 1% of the impurity phase. X-ray diffraction patterns were interpreted using Le Bail method (PCPDFWIN electronic catalog and JCPDS-ICDD database), and phase ratios were calculated using the Match! version 4.1 software. Volume fractions of the crystalline phase were determined with an error of ± 2%. All experiments were carried out at least 3 times (3–5 times). The results converged within the error limits. The errors fit within the symbol size in the presented data figures (see Figures 4, 5, 7, 10 and 12). During the analysis, the absence of partially oxidized phase (Pb_2_OF_2_) was additionally monitored based on the peak at 2Θ = 26.75° [11,29].

Energy-dispersive X-ray spectroscopy (EDS) microanalysis was performed to determine the actual composition of the samples using a scanning electron microscope VEGA3-LMU (TESCAN, Brno, Czech Republic) equipped with a lanthanum hexaboride thermionic cathode and an Oxford Instruments X-MAX-50 EDS detector, operating in the secondary electron mode. The AZTec software V.2.0 was utilized for data collection and processing. All measurements were conducted at room temperature, with no fewer than 8 points measured for each sample. Imaging was carried out at an accelerating voltage of 30 kV.

The impurity content of the starting materials and the resulting powders was determined by an inductively coupled plasma mass spectrometer NexION300D (PerkinElmer Inc., Waltham, MA, USA). Lead fluoride-based powders were dissolved in 20 mL of sulfuric acid (7N, 96%) purified by a surface distillation system Milestone DuoPUR (Milestone S.r.l., Sorisole, Italy) with microwave digestion of the sample in polytetrafluoroethylene autoclaves (DAP-100, PTFE, BERGHOFF GmbH&Co., Wenden, Germany) using a SPEEDWAVEFOUR microwave decomposition setup (BERGHOFF GmbH&Co., Wenden, Germany). The resulting solution was transferred to a polypropylene test tube and diluted with water. Deionized water was obtained using an Aquapuri 5–551 Series (Young Lin Instruments Co., Ltd., Hogye, Republic of Korea) and had an electrical resistance of 18.2 MΩ cm. The prepared solution was analyzed using inductively coupled plasma mass spectrometry (ICP-MS). Analytical measurements were carried out using an inductively coupled plasma mass spectrometer NexION300D (PerkinElmer Inc., Waltham, MA, USA) [29]. The resulting powders had a purity of greater than 99.99 wt% (see Table S1 for impurity analysis).

Europium (Eu^3+^) photoluminescence (PL) spectra were recorded using a Fluorolog FL3-22 spectrofluorometer (Horiba Jobin Yvon, Longjumeau, France) in the wavelength range of 400–700 nm with a 0.1 nm step, excited by a diode laser (λ = 377 nm).

Raman scattering spectra (RS) were investigated using a HORIBA LabRam Soleil instrument (Horiba Jobin Yvon GmbH, Bensheim, Germany) with the ability to excite RS with lasers of three wavelengths: 377 nm, 532 nm, and 785 nm. In our case, only the 785 nm excitation wavelength was used, since others caused the excitation of Eu^3+^ fluorescence [30]. Measurements were carried out in the range of 149–3500 cm^−1^ with a 2 cm^−1^ step.

Infrared (IR) transmittance measurements were performed using a Tensor 27 Fourier-transform infrared spectrometer (FTIR) (Bruker Optics Inc., Billerica, MA, USA) in the range of 400–8000 cm^−1^ with a 1.9 cm^−1^ step.

## 3. Results and Discussion

### 3.1. Position of the Solvus Line on the PbF_2_-EuF_3_ Diagram

**Figure 2:**
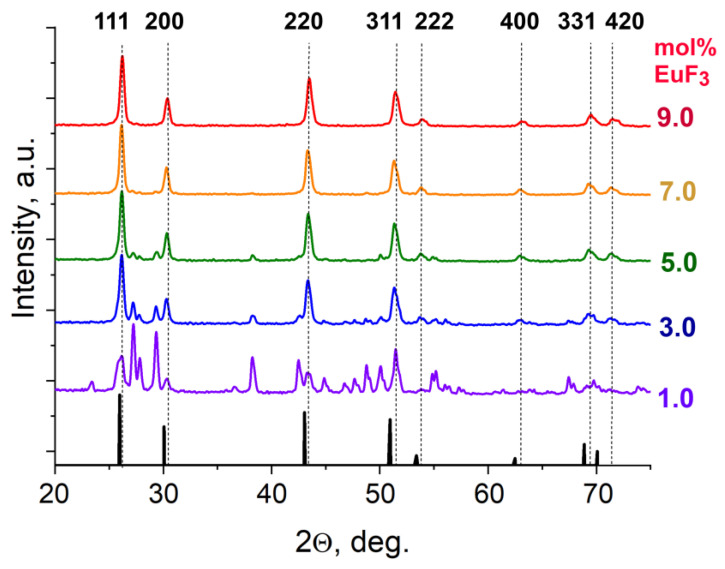
X-ray diffraction patterns of the (1 − *x*)(PbF_2_)x(EuF_3_) samples with 1–9 mol% EuF_3_ nominal content. Dot lines and Miller indices indicate the reflections of the cubic solid-solution phase, and black bars are reference data for pure cubic β-PbF_2_ according to COD-1530196 [31].

The samples synthesized by the co-precipitation method have appeared as fine white powders. The appearance did not change depending on the nominal europium content, indirectly indicating the absence of oxidized phases. Analysis of the phase composition of the samples after synthesis at nominal concentrations from 0.5% to 7 mol% EuF_3_ revealed the presence of two phases simultaneously: α-PbF_2_ and cubic Pb_1−x_Eu_x_F_2+x_. From 8 to 12 mol%, there was only one cubic phase of the solid solution with parameters corresponding to the nominal composition (Figure 2). In 0–0.5 mol% EuF_3_ composition range, the X-ray reflections belonging to the rhombic phase corresponded to pure α-PbF_2_.

It is worth noting that the reflections of the cubic solid solution Pb_1−x_Eu_x_F_2+x_ are shifted relative to the reflections of nominally pure cubic β-PbF_2_ at 2Θ large angles, which indicates the compression of cubic lattice parameters because of the incorporation of smaller Eu^3+^ ions into a solid-solution crystal lattice.

The lattice parameter for these solid solutions obeys a linear equation (Vegard’s law):

*a* = 5.940 + *k_Eu_·x*, (3)

where 5.940 Å—the β-PbF_2_ lattice parameter; *x*—the mol% of RE; and the coefficient *k*_Eu_ = −0.00237 as determined in [10,11].

The lattice parameter of the obtained solid solutions in the range of nominal EuF_3_ content from 1 to 7 mol% was found to be 5.919 ± 0.003 Å, corresponding to the EuF3 content of 7.0–9.5 mol% according to the Vegard law.

**Figure 3:**
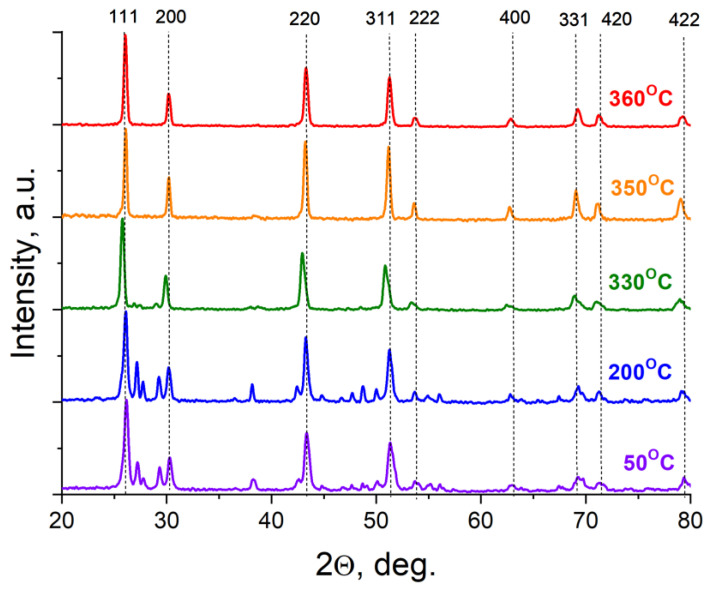
X-ray diffraction patterns of Pb_0.97_Eu_0.03_F_2.03_ samples after annealing at different temperatures (dashed lines and Miller indices indicate reflections of the cubic solid-solution phase).

The examination of the PXRD patterns after heat treatments revealed (Figure 3, Figure S1, Table S2 for the volume fraction of the cubic phase) that the proportion of the cubic phase began to increase starting from 300 °C. For the sample with a nominal composition of Pb_0.97_Eu_0.03_F_2.03_, at 330 °C, the volume fraction of α-PbF_2_ is small compared to a solid-solution cubic phase (Figure 3), which is consistent with the findings of a previous study [10].

**Figure 4:**
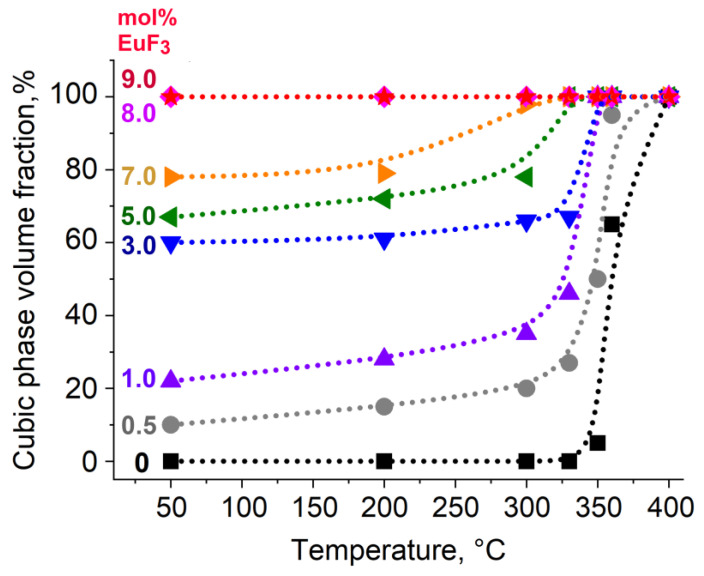
Dependence of cubic phase volume fraction on temperature for (1 − *x*)(PbF_2_)x(EuF_3_) samples.

A sharp increase in the proportion of the cubic phase is observed at temperatures above 330 °C; samples with a nominal EuF_3_ content exceeding 5 mol% are already single-phased in this temperature range. In nominally pure PbF_2_, the rhombic phase is detected even after heat treatment at 360 °C (Figure 4).

**Figure 5:**
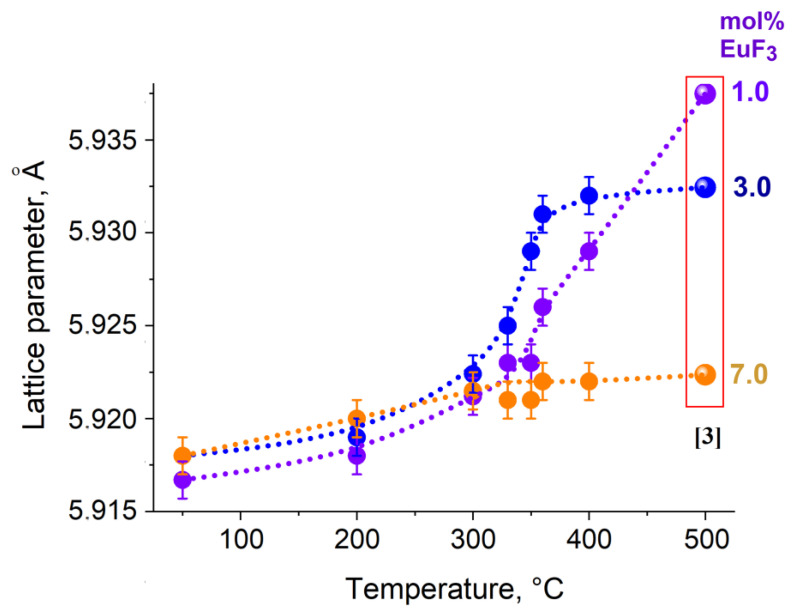
Variation in lattice parameters of the cubic phase after heat treatment of samples with nominal composition Pb_1−x_Eu_x_F_2+x_.

Remarkably, the lattice parameters of the cubic phase after heat treatment show a tendency to increase (Figure 5), indicating an approach of the lattice parameters of solid solutions obtained by solid-phase synthesis at 500 °C [11].

Such lattice parameter behavior and the unchanged properties of the powders after an additional 24 h heat treatment indicate that the obtained values are close to equilibrium state.

Eu^3+^ is a luminescent-active impurity, and its spectrum is sensitive to changes in the symmetry of the surrounding environment. Thanks to this, the Eu^3+^ ion is often used as a spectroscopic probe, making samples with Eu^3+^ concentration of 0.1 mol% [31]. It is considered that, at such concentrations, the additional introduction of Eu does not significantly alter the structure. In our case, the Eu concentration is knowingly higher and varies from sample to sample, so the use of all the fine capabilities of spectroscopic probing analysis may be incorrect; hence, we used only an estimation of local symmetry by the asymmetry coefficient [11,12].

**Figure 6:**
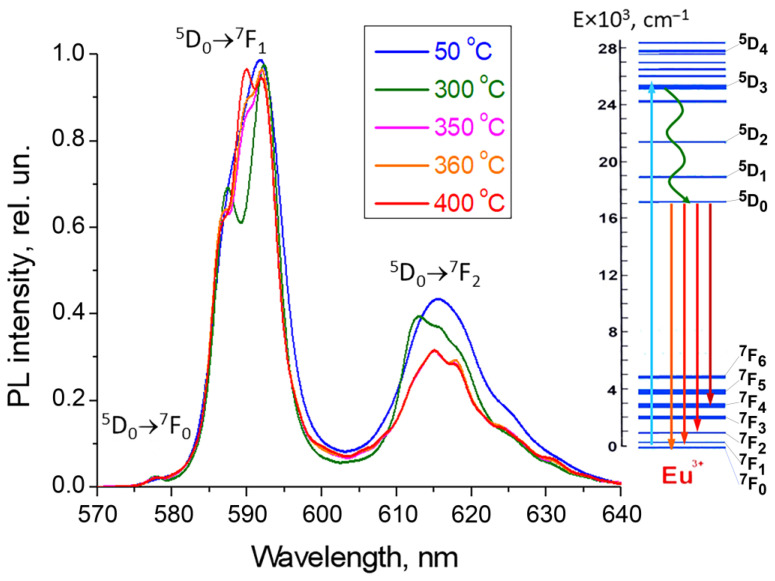
Normalized PL spectra of Pb_0.97_Eu_0.03_F_2.03_ solid solutions after heat treatment at different temperatures ^(^λ^exc^ = 377 nm). The inset shows a diagram of the Eu^3+^ levels.

The electric dipole transition ^5^D_0_ → ^7^F_2_ (~ 612 nm) in the Eu^3+^ ion is highly sensitive. The magnetic dipole transition ^5^D_0_ → ^7^F_1_ (~ 590 nm), permitted in terms of parity, has an intensity that is practically independent of the point symmetry of the luminescent center and its environment. To characterize the local environment of Eu^3+^ ions, the asymmetry coefficient R21 is used. By definition, it is the ratio of the intensities of the highly sensitive electric dipole transition ^5^D_0_ → ^7^F_2_ and the magnetic dipole transition ^5^D_0_ → ^7^F_1_ [12]:

(4) R21= IEDD50→F72IMDD50→F71,

The greater the value of this ratio, the less symmetrical the environment of the europium ion in this matrix. Dominance in the intensity of the band corresponding to the magnetic dipole transition ^5^D_0_ → ^7^F_1_ over the electric dipole transition ^5^D_0_ → ^7^F_2_ indicates an environment of the Eu^3+^ ion that is close to centrosymmetric, as observed in solid solutions (Figure 6).

The transition between the energy levels ^5^D_0_ and ^7^F_0_ is also forbidden by the selection rules for electronic dipole transitions; moreover, these levels are degenerate and therefore have zero Stark splitting. This transition is practically absent in the fluorescence spectra of solid solutions, confirming a symmetry close to cubic for the Eu^3+^ optical centers.

**Figure 7:**
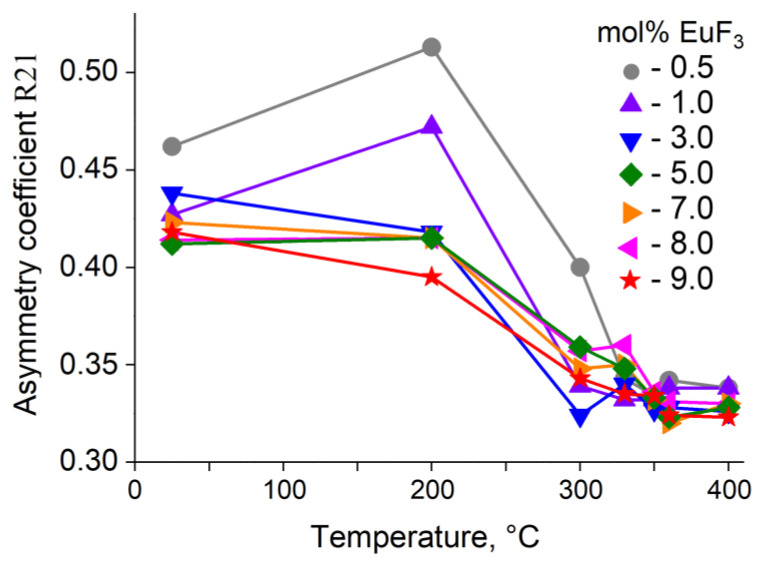
Changes in the asymmetry coefficient after the annealing at different temperatures of samples with a nominal composition of Pb_1−x_Eu_x_F_2+x_.

As the annealing temperature increases, the asymmetry coefficient behaves differently depending on the nominal composition (Figure 7).

In samples with a nominal content of EuF_3_ ranging from 3 to 9 mol%, there is a gradual decrease in R21, indicating an increase in symmetry. In samples with 0.5 and 1 mol% EuF_3_, initially up to 200 °C, R21 increases due to structure relaxation, followed by a sharp decrease in R21, indicating increased centrosymmetry. In the phase transition region, R21 in all samples ranges from 0.30 to 0.35 (see Table S3 for values of the asymmetry coefficient R21). Thus, the structural data obtained from XRD and luminescence studies coincide.

**Figure 8:**
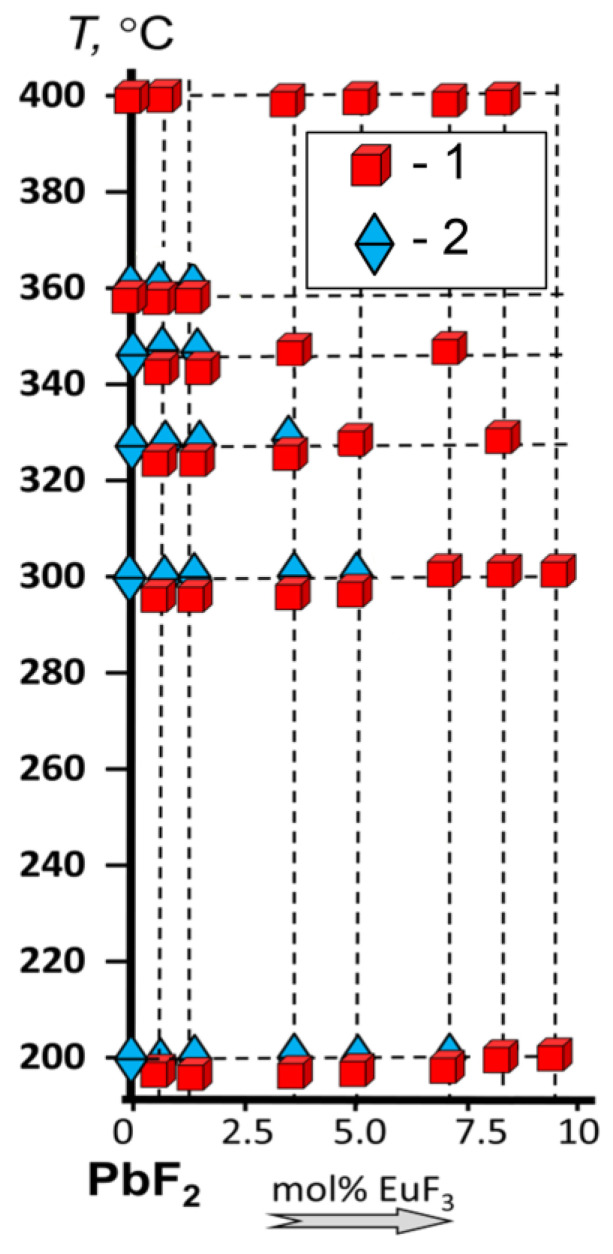
Fragment of the T-X diagram of the quasi-binary system PbF_2_-EuF_3_, according to the results of XRD analysis: 1—cubic phase (*Fm*$\bar{3}$*m*); 2—rhombic phase (*Pnma*).

Figure 8 schematically represents a fragment of the T-X diagram of the quasi-binary system PbF_2_-EuF_3_ in the investigated range of temperatures and compositions. It is evident that, with the increase in the nominal content of EuF_3_, the temperature of the phase transition sharply decreases. It is worth noting that the phase transition in all investigated compositions is “frozen”, as in nominally pure PbF_2_ [9], meaning that no phase separation occurs upon cooling for a long time (at least, no changes in the structure and luminescence of annealed powders have occurred over one year of exposition. See Figure S3).

### 3.2. Determining the Existence Region of the Rhombohedral R-Phase in the PbF_2_-EuF_3_ System

To determine the existence of the R-phase, samples were synthesized in the nominal Eu concentration range of 35–45 at% using the co-precipitation and solid-phase methods. In both cases, the synthesized samples appeared as white powders. It was particularly important in the case solid-phase synthesis, indicating the absence of oxidation processes and the formation of lead oxyfluorides and oxides.

X-ray spectral microanalysis allowed determining that the deviations from the theoretically calculated compositions of the samples during synthesis do not exceed 0.05 at% (see Table S4 for SEM-EDX analysis). The SEM analysis of powder images shows that the particle sizes obtained by the co-precipitation technique vary widely from several to tens of microns, with irregular shapes (Table S4). Finer powders (ranging from 0.5 to 1 micron) were obtained through solid-phase synthesis, attributed to several grinding steps during synthesis.

**Figure 9:**
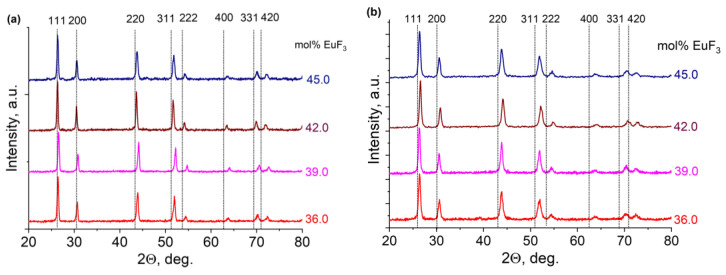
X-ray diffraction patterns of samples obtained by the solid-phase synthesis (**a**) and co-precipitation technique (**b**) in the quasi-binary system PbF_2_-EuF_3_ (dashed lines and Miller indices indicate nominally pure β-PbF_2_ according to COD-1530196 [31]).

When studying the diffraction patterns of samples obtained by solid-phase synthesis (Figure 9a) and co-precipitation from aqueous nitrate solutions (Figure 9b), the presence of solid solutions with parameters different from the high-temperature cubic β-PbF_2_ was confirmed, which was especially noticeable at 2Θ large values. It results from lattice parameters.

To confirm the assumption of the presence of the R-phase, the calculation of the unit cell parameter was carried out based on the obtained XRD data. Initially, the peaks were indexed within the framework of a cubic cell (*Fm*$\bar{3}$*m*) with parameters close to the high-temperature modification of lead fluoride. The obtained values of the unit cell parameter for samples obtained by solid-phase synthesis and the co-precipitation technique are presented in Table S5.

**Figure 10:**
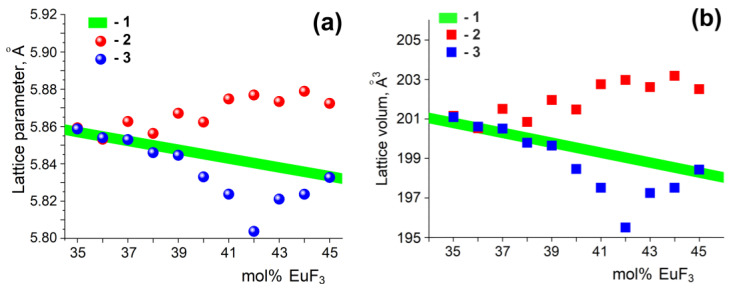
Lattice parameter (**a**) and lattice volume (**b**) for solid-solution (100 − x)PbF_2−x_EuF_3_ (x = 35–45 mol% EuF_3_) samples obtained by the co-precipitation (3) and solid-phase methods (2) and calculated by Vegard’s law (1–green line).

Graphically analyzing the obtained results (Figure 10a), a significant difference is noticeable between the theoretical values (green line in Figure 10) obtained according to Vegard’s law for a hypothetical solid solution Pb_1−x_Eu_x_F_2+x_ and the values of the unit cell parameter determined for samples synthesized by the co-precipitation and solid-phase methods. According to [32], the deviation is better observed in terms of the volume of the crystalline cell (Figure 10b), and correspondingly in linear equations (Röttgers’s rule):

*V* = *V*_0_ + *k_V_·x*, (5)

where *V*_0_—the unit cell volume of the fluorite matrix MF_2_ (for β-PbF_2_ 209,58 Å^3^); *x*—the mol% of RE; *k_V_*—the coefficient.

*k_V_* = 3*a*_0_k, (6)

Thus, from Equations (3) and (6), *k_V_*_Eu_ = −0.2509.

In the range of 40–45 mol% EuF_3_, deviations in cell parameter and volume from the calculated values are observed. Scanning with a larger step [11] indicated that the violation of Vegard’s law begins at 36 ± 2 mol% EuF_3_, but a significantly different phase, based on the rhombic (*Pnma*) modification of europium trifluoride Er_1-y_Pb_y_F_3-y_, can only be observed starting from 60 mol% EuF_3_. Thus, deviations from linear laws in the 40–45 mol% EuF_3_ range may be associated with the existence of a phase in this range, with reflections close to the Pb_1−x_Eu_x_F_2+x_ cubic solid solution, i.e., the R-phase.

We discovered that deviations from the linear law for samples obtained by solid-phase synthesis and co-precipitation were in opposite directions. A slight increase in the cell parameter for solid-phase samples corresponding to a solid solution with lower Eu concentration may indicate the presence of a narrow two-phase equilibrium region of the cubic solid solution Pb_1−x_Eu_x_F_2+x_ and its distorted modification, the R-phase. A decrease in the cell parameter for samples obtained by the co-precipitation technique may be related to the non-equilibrium nature of the obtained phases.

**Figure 11:**
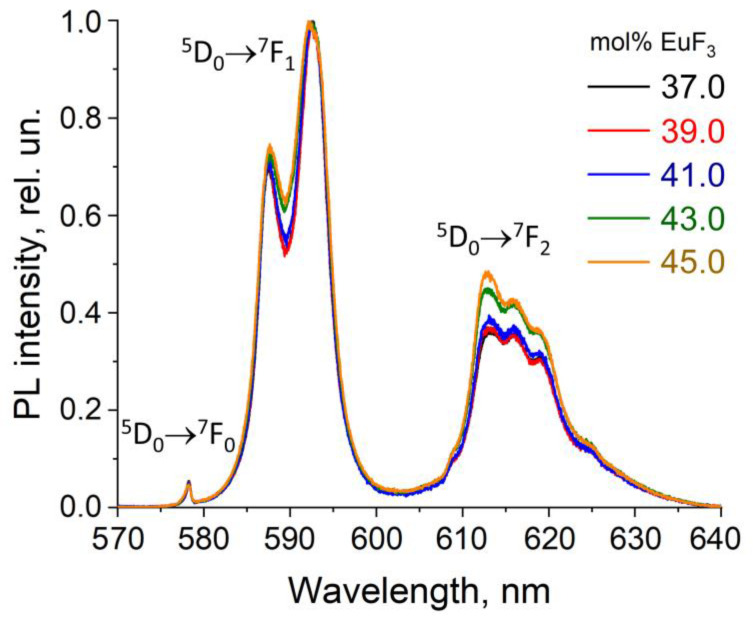
Normalized PL spectra of samples in the (100 − x)PbF_2−x_EuF_3_ system (x = 37–45 mol% EuF_3_), obtained by the co-precipitation technique (λ^exc^ = 377 nm).

The fluorescence spectra of powder samples in this concentration range were also investigated (Figure 11).

**Figure 12:**
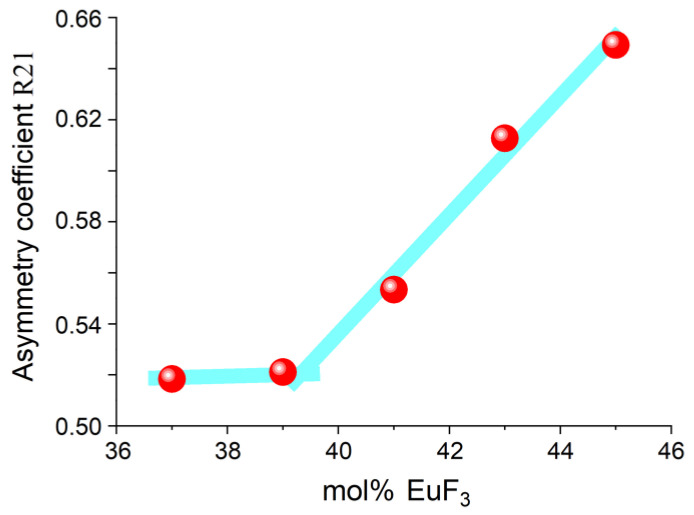
Dependence of the asymmetry coefficient R21 on the EuF_3_ content in the system (100 − x)PbF_2−x_EuF_3_ (x = 37–45 mol% EuF_3_), obtained by the co-precipitation technique.

In 37–45 mol% EuF_3_ concentration range, the asymmetry coefficient was significantly higher than that in the range up to 10 mol% EuF_3_. For samples with composition (100 − x)PbF_2−x_EuF_3_ (x = 41–45 mol% EuF_3_), the coefficient R21 gradually increases (see Figure 12), indicating a slight increase in asymmetry due to the heterovalent substitution in the solid solution. However, with further increase in Eu concentration, R21 undergoes a sharp increase. Thus, the boundary at which the local structure begins to distort significantly is approximately 39 mol% Eu.

Additionally, vibrational spectra were investigated in this system. The complete vibrational representation for crystals with a fluorite structure can be expressed as follows [33]:

Γ = F_1u_ (IR) + F_2g_ (R), (7)

where F_1u_ represents a triply degenerate, antisymmetric vibration with respect to the center of symmetry, active in the infrared spectrum, and F_2g_ represents a triply degenerate, symmetric vibration with respect to the center of symmetry, active in the Raman spectrum.

The FTIR spectroscopy (Figure S2) in our case does not allow determining the vibrational modes of cubic PbF_2_, as the F_1u_ vibrations lie around 347 cm^−1^ (less than 400 cm^−1^) [34]. However, it did reveal the presence of nitro groups [NO_3_]^−^ [35] and hydroxyl groups [OH]^−^ [35] in samples obtained by co-precipitation (see Figure S2, and Table S6 for absorption spectra details), indicating the high sensitivity of the method, as the qualitative reaction of diphenylamine was negative, and the samples were dried for an extended period.

**Figure 13:**
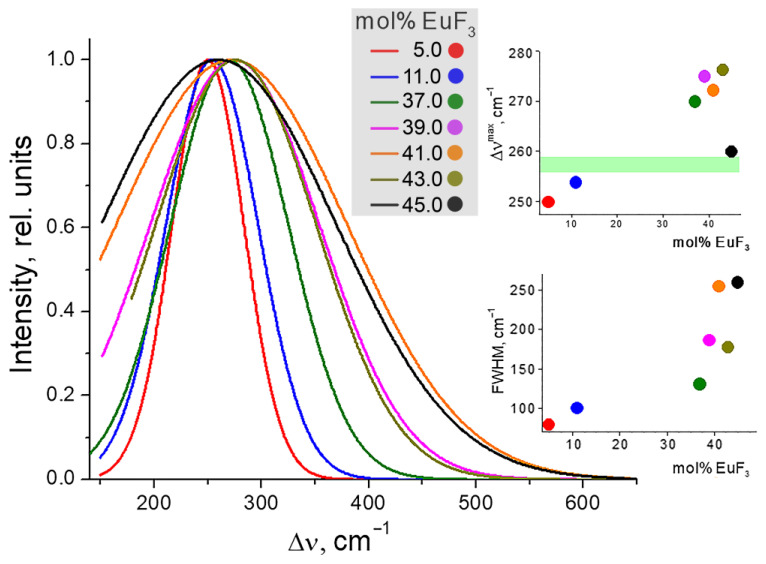
Overall view of the normalized and Gaussian-fitted Raman spectra of (100 − x)PbF_2−x_EuF_3_ samples (x = 37–45 mol% EuF_3_), obtained by the co-precipitation technique. The insertions show the dependences of Δν^max^ and FWHM vs. EuF_3_ concentration. The green line is attributed with the nominal pure cubic β-PbF_2_ [33].

In the Raman spectra of crystals of undistorted fluorite-type, a single line will be observed, and the frequency of this line for a crystal of nominally pure cubic β-PbF_2_ falls within the range of 256–259 cm^−1^ [36], corresponding to the F_2g_ vibration mode. This is clearly seen in the spectra of single-phase samples with concentrations of 5 and 11 mol% EuF_3_. When comparing the Raman spectra of samples with Eu concentrations ranging from 37 to 45 mol% EuF_3_, a noticeable shift in the peak maximum and broadening of the peaks can be observed (Figure 13, see Table S7 for Raman spectra analysis). The Raman spectra present a superposition of one cubic phase maximum and several R-phase maxima. Since the R-phase is a rhombic deformed cubic phase with similar lattice parameters, its Raman spectrum represents the broadening and splitting of one cubic phase band, as shown for the Ba4Y3F17 phase in a similar system [37]. Thus, a sharp broadening of the Raman band with a shift to the region of high energies indicates the formation of the R-phase.

One can observe a significant shift in the peak maximum for samples containing Eu 37–43 at%, indicating the presence of another phase structurally similar (Figure 13). Since the phase of the cubic solid solution Pb_1−x_Eu_x_F_2+x_ and the R-phase are very close in structure and parameters, the splitting of the band into close components leads to its broadening. The narrowing of the band in the region of 43 at% Eu may be associated with the crystallization region of only the R-phase.

**Figure 14:**
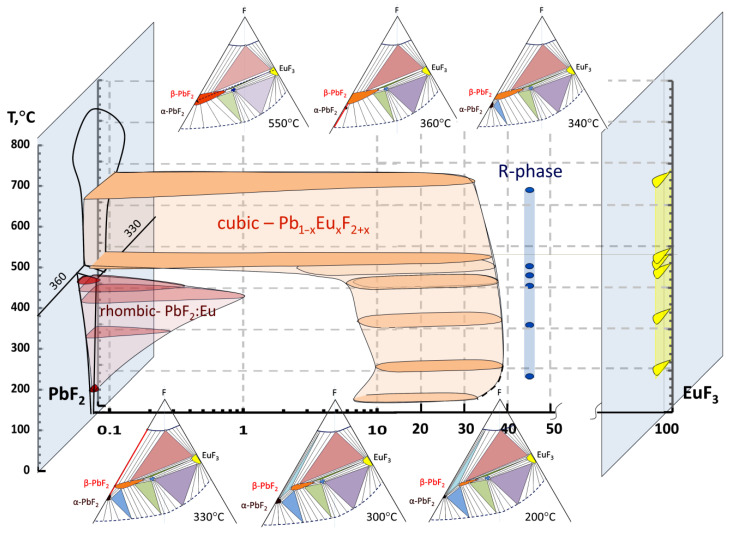
T-X diagram near quasi-binary PbF_2_-EuF_3_ section of ternary Pb-Eu-F system in variable scale. The sketches of isothermal cross-sections of T-X-Y projection of ternary Pb-Eu-F diagram are presented schematically.

Summarizing the results of phase equilibria analysis, we reconstructed the T-X-Y diagram of Pb-Eu-F ternary system within the PbF_2_-EuF_3_ section (Figure 14). In this study, we proved the general view of T-X-Y diagram [11] but expanded the temperature range from 50 to 550 °C and clarified the concentration ranges of homogeneity limits for cubic and rhombic PbF_2_:Eu and R-phase. For better understanding, we presented the fragment of T-X-Y diagram nearby the PbF_2_-EuF_3_ quasi-binary cross-section in a variable scale with the large logarithmic scale near PbF_2_ and the linear scale starting from 10 mol% EuF_3_. The homogeneity limits were determined along the PbF_2_-EuF_3_ quasi-binary cross-section in mol% EuF_3_. Their widths towards the Metal-F direction were less than 1 mol%, which is typical for the homogeneity ranges of fluoride binary compounds [9]. Also, cross-sections of T-X-Y projection of ternary Pb-Eu-F diagram are presented schematically for certain temperatures. For better understanding, the areas of trivariant equilibria, i.e., phase homogeneity areas, are presented on a large scale, contrary to the common view when they presented as dots on the actual scale.

The main features of the analyzed system were determined, and it was established that the homogeneity region of the R-phase is about 1 mol.% and spans 44–45 mol% EuF_3_, slightly changing with temperature over the entire temperature range under consideration.

The cubic solid solution Pb_1−x_Eu_x_F_2+x_ spans 7.5–38 mol% EuF_3_ at 50–200 °C with a possible retrograde solvus behavior at low temperatures (dash line in Figure 14). It then expands from 7.5 to 37 mol% EuF_3_ at 300 °C. Starting at 330 °C, it reaches the T-Pb-F plane and no longer deviates from it with increasing temperature. The Eu-rich boundary of the cubic solid solution Pb_1−x_Eu_x_F_2+x_ shifts from 37 to 32 mol% EuF_3_ at 550 °C.

Simultaneously, PbF_2_:Eu rhombic phase expanded from 0.1 to 1 mol% EuF_3_ at a temperature rise from 50 to 280 °C. After then, it is quickly compressed to zero at 360 °C. In the entire temperature range under consideration, the homogeneity region of the orthorhombic PbF_2_ phase does not detach from the T-Pb-F plane.

So, we established that the stable cubic phase in PbF_2_-EuF_3_ quasi-binary system could be obtained at all temperatures starting from RT to 550 °C, and it exists in a wide concentration range up to 38 mol% of EuF_3_.

**Figure 15:**
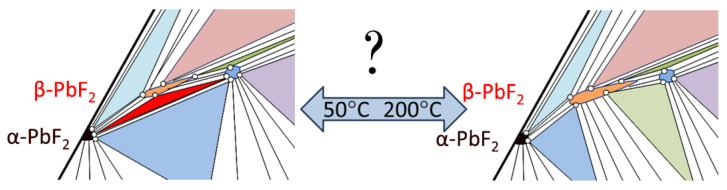
Fragments of the possible isothermal cross-sections of the T-X-Y projection of the ternary Pb-Eu-F diagram in the 50–200 °C temperature range.

More detail bivariant and monovariant equilibria with Pb_1−x_Eu_x_F_2+x_ cubic solid-solution phase and R-phase are plotted in Figure 15 for the T-X-Y cross-section at 200 °C. We can see S_cubic solid solution_S_R-phase_V bivariant equilibrium in 38–44 mol% EuF_3_ range, which slightly broadens with an increase in temperature (see Figure 14).

The only currently unclear issue is the existence of S_α-PbF2_S_β-PbF2_S_R-phase_V monovariant equilibrium at low temperatures (Figure 15—red triangle). We believe this issue is important for the future operation of devices based on a cubic solid solution Pb_1−x_Eu_x_F_2+x_ phase, but for successful design of materials based on the cubic phase, detailed investigations of 3D homogeneity range must be carried out. And the first challenge in these investigations is having a method of measurement of nonstoichiometry for fluorine similar to those used for oxygen for the high-temperature superconducting YBa_2_Cu_3_O_7-δ_ phase [38].

## 4. Conclusions

Fluoride solid solutions in lead–europium systems are of significant interest for optical and electronic applications due to their unique luminescent properties and structural characteristics. Moreover, understanding phase relationships and structural transitions in these systems is crucial for optimizing their functional properties. However, the complete phase diagram and structural behavior across the full composition range of the (100 − x)PbF_2−x_EuF_3_ quasi-binary system remains incompletely characterized, particularly regarding phase transition mechanisms and local structural environments. Therefore, we conducted a comprehensive investigation of phase relationships and structural transitions across two distinct composition ranges of the (100 − x)PbF_2−x_EuF_3_ system using multiple synthesis and characterization approaches.

For the low-europium range (x = 0–12 mol% EuF_3_), samples were synthesized by co-precipitation from aqueous solutions. Phase composition analysis revealed pure α-PbF_2_ and cubic solid solution Pb_0.93_Eu_0.07_F_2.07_ for x = 0.5–7 mol% and cubic solid solution Pb_0.93_Eu_0.07_F_2.07_ for x = 8–12 mol%. Thermal treatments at 200–400 °C enabled the determination of phase transition temperatures for samples with x = 0–7 mol%. Investigation of Eu^3+^ local structure showed significant symmetry increases during thermal treatments at 300–350 °C, allowing solvus line position determination.

For the high-europium range (x = 35 to −45 mol% EuF_3_), samples were synthesized using both solid-phase and liquid-phase methods. X-ray diffraction analysis revealed deviations from Vegard’s law and Röttgers’s rule in the 39–44 mol% Eu range. Spectral-luminescent analysis co-precipitated samples (37–44 mol% EuF_3_) showed significant changes in asymmetry coefficient R21, indicating altered europium ion environment symmetry. Raman spectroscopy confirmed a phase slightly different from the cubic structure in the 37–43 at% Eu range. The results establish that two close phases coexist in the x = 36–42 mol% EuF_3_ range: cubic Pb_0.64_Eu_0.36_F_2.36_ and rhombically distorted R-phase. At 43 mol% EuF_3_, only the R-phase appears to be present, evidenced by Raman band narrowing, while higher EuF_3_ concentrations establish phase equilibrium among R-phase, Eu_1−γ_Pb_γ_F_3−γ_, and EuF_3_.

## Data Availability

The original contributions presented in this study are included in the article/Supplementary Materials. Further inquiries can be directed to the corresponding authors.

## Supplementary Materials

The following supporting information can be downloaded at: https://www.mdpi.com/article/10.3390/ma19010195/s1, Figure S1: X-ray powder diffraction patterns of sample synthesized in the PbF_2_-EuF_3_ quasi-binary system by the co-precipitation technique and annealed at different temperatures; Figure S2: Fragment of the IR transmittance spectra of samples in the system (100 − x)PbF_2−x_EuF_3_ (x = 35–45 mol.% EuF3), obtained by the co-precipitation technique; Figure S3: PL spectra of as-synthesized samples and after 1-year exposition under normal RT conditions; Table S1: Impurity element concentrations in the samples of PbF_2_ and Pb_0.65_Eu_0.35_F_2.35_ solid solution determined by ICM-MS; Table S2: Volume fraction of the cubic phase during heat treatment; Table S3: Values of the asymmetry coefficient R21 for samples with and without heat treatment; Table S4: Results of scanning electron microscopy supported by EDS analysis; Table S5: Cell parameter of Pb_1−x_Eu_x_F_2+x_ samples (x = 35–45 mol%); Table S6: Absorption band values for (100 − x)PbF_2−x_EuF_3_ samples (x = 35–45 mol.% EuF_3_); Table S7: Raman spectra of solid solutions with different Eu content.
