# Supplementary material for: Fundamentals of Cubic Phase Synthesis in PbF2–EuF3 System
Source: Materials (Basel). 2026 Jan 5;19(1):195. doi: 10.3390/ma19010195 (PMC12786506; doi:10.3390/ma19010195)
Supplement: Supplementary file 1 [file materials-19-00195-s001.zip › Supplementary.pdf]

Supplementary Materials

Fundamentals of cubic phase synthesis in PbF_2 - EuF_3 system

Sofia Zykova, Kristina Runina, Mariya Mayakova, Maria Berezina, Olga Petrova, Roman Avetisov, and Igor Avetissov

Table S1. Impurity element concentrations in the samples of PbF_2 and $Pb_{0.65}Eu_{0.35}F_{2.35}$ solid solution determined by ICM-MS.

Element	PbF_2	$Pb_{0.65}Eu_{0.35}F_{2.35}$
	wt%	wt%
Li	< 7.59E-07	< 7.15E-07
Be	< 3.15E-07	< 1.50E-07
B	< 2.06E-05	< 3.00E-07
Na	< 6.56E-05	< 6.34E-05
Mg	< 2.00E-05	< 7.24E-05
Al	5.30E-05	2.56E-04
Si	1.56E-03	4.92E-03
K	< 9.87E-05	< 6.46E-04
Ca	< 8.68E-05	< 5.34E-04
Sc	< 1.12E-06	3.56E-06
Ti	< 1.73E-06	1.25E-05
V	< 1.31E-05	< 3.31E-05
Cr	3.38E-06	4.87E-05
Mn	< 4.45E-06	8.37E-05
Fe	3.80E-05	3.89E-05
Co	< 6.77E-06	2.13E-06
Ni	< 9.60E-07	7.77E-06
Cu	< 3.60E-06	4.45E-05
Zn	< 3.68E-05	< 6.36E-05
Ga	< 9.02E-06	< 2.06E-05
Ge	< 8.17E-05	< 5.76E-05
As	1.92E-05	3.29E-04
Se	< 2.09E-05	9.63E-05
Rb	< 2.35E-06	< 4.13E-06
Sr	< 1.33E-06	6.13E-06
Y	< 4.78E-07	3.42E-06
Zr	< 6.56E-07	< 2.46E-06
Nb	< 5.77E-06	< 9.07E-06
Mo	< 2.03E-05	< 5.82E-06
Ru	< 3.18E-07	8.06E-07
Rh	Pb++	Pb++
Pd	< 2.85E-07	< 2.55E-07
Ag	< 6.31E-07	7.31E-04

Element	PbF_2	$Pb_{0.65}Eu_{0.35}F_{2.35}$
	wt%	wt%
Cd	< 3.55E-05	< 2.07E-05
In	< 2.10E-08	< 4.50E-08
Sn	< 4.17E-06	< 3.28E-05
Sb	< 1.73E-06	1.47E-06
Te	< 3.86E-05	< 4.08E-05
Cs	3.44E-06	< 9.92E-07
Ba	1.98E-05	1.71E-05
La	5.54E-06	6.50E-05
Ce	8.04E-07	1.35E-06
Pr	1.01E-04	< 2.16E-07
Nd	< 2.26E-06	4.56E-07
Sm	< 5.25E-08	< 6.00E-08
Eu	< 5.25E-08	matrix
Gd	1.75E-06	< 1.05E-06
Tb	2.02E-06	< 3.14E-07
Dy	< 6.30E-08	< 4.50E-08
Ho	6.44E-07	2.48E-07
Er	< 1.05E-08	1.83E-07
Tm	< 4.12E-08	< 3.00E-08
Yb	7.47E-06	5.98E-06
Lu	< 1.03E-07	2.84E-07
Hf	< 1.05E-08	3.30E-07
Ta	< 1.05E-08	< 1.49E-06
W	2.42E-05	4.21E-06
Re	< 3.15E-08	< 4.50E-08
Os	< 3.15E-08	< 4.50E-08
Ir	< 3.15E-08	< 3.00E-08
Pt	< 6.64E-06	1.36E-05
Au	1.54E-06	< 7.51E-08
Hg	2.44E-05	< 8.23E-06
Tl	< 4.31E-06	8.44E-07
Pb	matrix	matrix
Bi	1.66E-04	1.27E-04
Th	< 1.29E-06	< 6.25E-08
U	< 3.98E-08	4.92E-08

Total sum	2.63E-03	8.44E-03
Purity, wt%	99.99737	99.9916

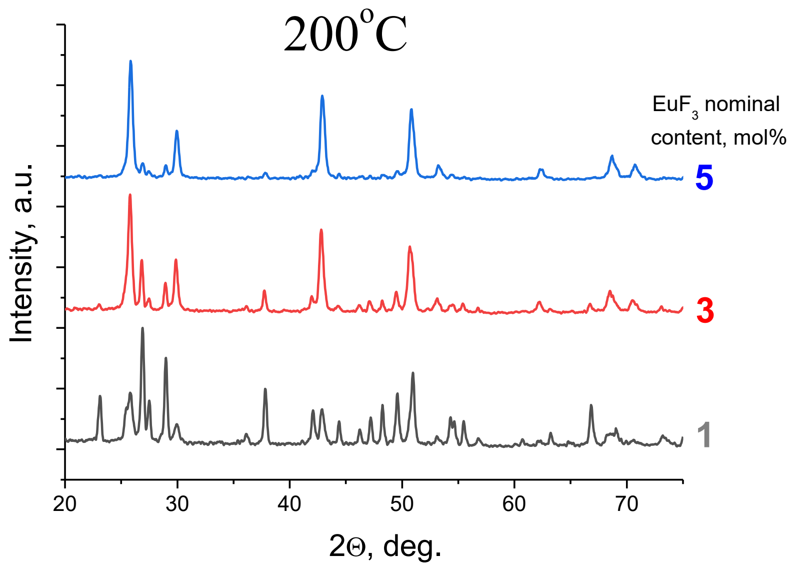
200°C
EuF3 nominal content, mol%
5
3
1
Intensity, a.u.
20
30
40
50
60
70
2Θ, deg.

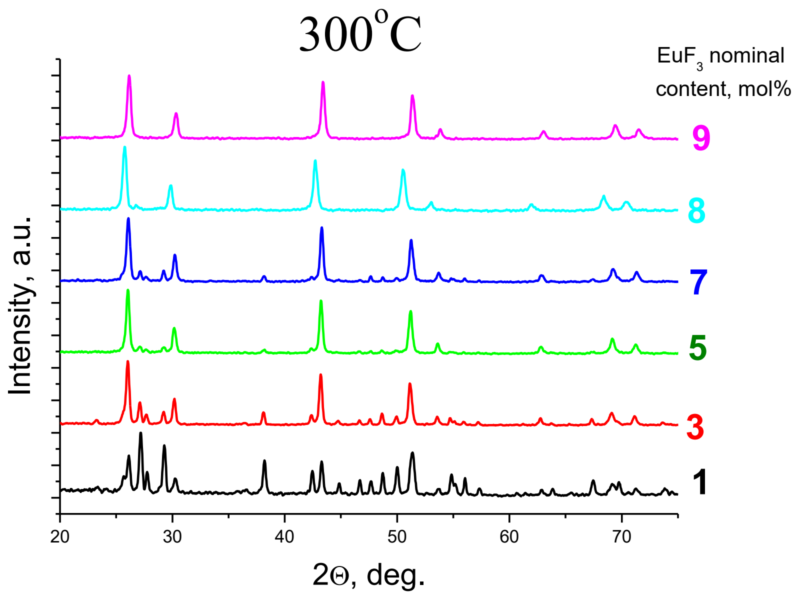
300°C
EuF3 nominal content, mol%
9
8
7
5
3
1
Intensity, a.u.
20
30
40
50
60
70
2Θ, deg.

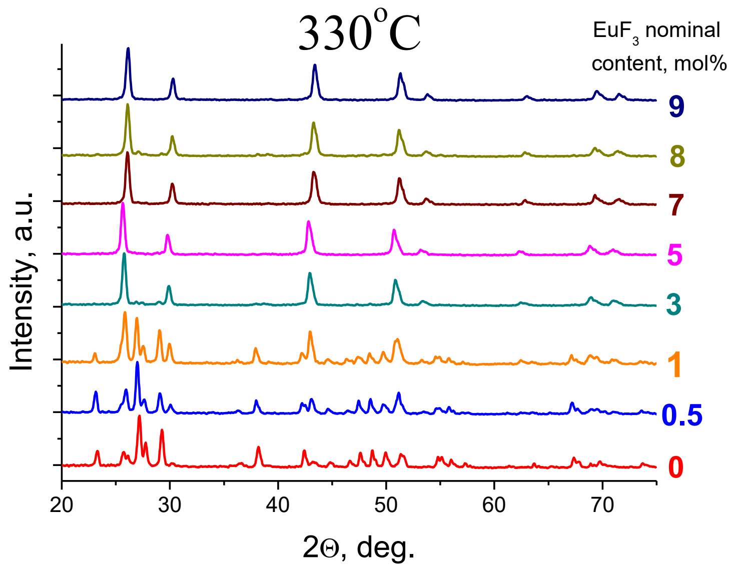
330°C
EuF3 nominal content, mol%
9
8
7
5
3
1
0.5
0
Intensity, a.u.
20
30
40
50
60
70
2Θ, deg.

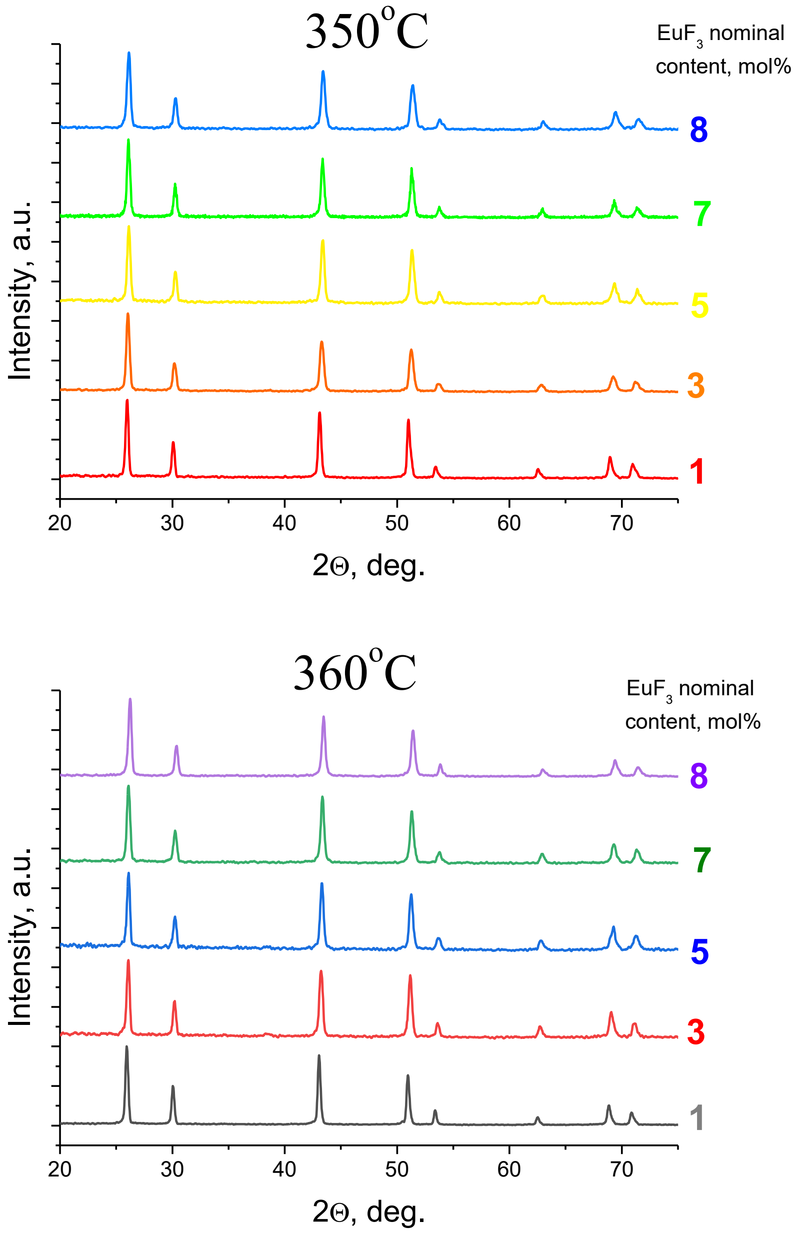

Figure S1. X-ray powder diffraction patterns of sample synthesized in the PbF_2-EuF_3 quasi-binary system by the co-precipitation technique and annealed at different temperatures.

Table S2. Volume fraction of the cubic phase during heat treatment

T, °C	Nominal content of Eu, mol%							
	0	0.5	1	3	5	7	8	9
50	0	10	22	60	67	78	100	100
200	0	15	28	61	72	79	100	100
300	0	20	35	66	78	98	100	100
330	0	27	46	67	100	100	100	100
350	5	50	100	100	100	100	100	100
360	65	95	100	100	100	100	100	100
400	100	100	100	100	100	100	100	100

Table S3. Values of the asymmetry coefficient R21 for samples with and without heat treatment.

Nominal content of Eu, mol%	Ratio ED/MD ($^5D_0$–$^7F_2$ /$^5D_0$–$^7F_1$)						
	Powders						
	50	200	300	330	350	360	400
0.5	0.462	0.513	0.400	0.343	0.334	0.342	0.338
1	0.427	0.472	0.339	0.332	0.332	0.338	0.338
3	0.438	0.418	0.324	0.340	0.327	0.328	0.336
5	0.414	0.415	0.357	0.360	0.336	0.331	0.334
7	0.423	0.415	0.348	0.350	0.332	0.320	0.330
8	0.412	0.415	0.359	0.348	0.333	0.323	0.328
9	0.418	0.395	0.343	0.335	0.334	0.324	0.323

Table S4. Results of scanning electron microscopy supported by EDS analysis.

Nominal composition at%	Co-precipitation technique		Solid-phase synthesis	
	Composition by analysis, at%	SEM image	Composition by analysis, at%	SEM image
$Pb_{0.64}Eu_{0.36}F_{2.36}$			Pb<br>0.64±0.05<br><br>Eu<br>0.36±0.05<br><br>F<br>2.36±0.27	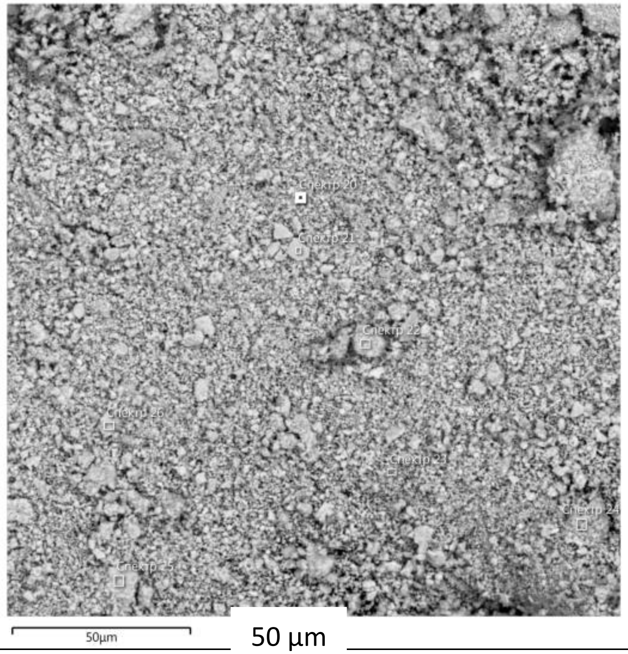

$Pb_{0.62}Eu_{0.38}F_{2.38}$	Pb 0.62±0.04<br><br>Eu<br>0.38±0.02<br><br>F<br>2.39±0.15	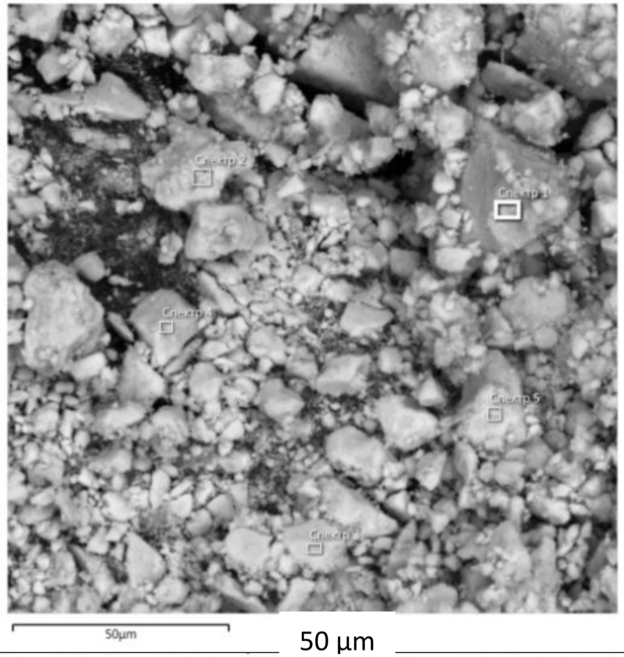	Pb<br>0.61±0.02<br><br>Eu<br>0.39±0.04<br><br>F<br>2.49±0.20	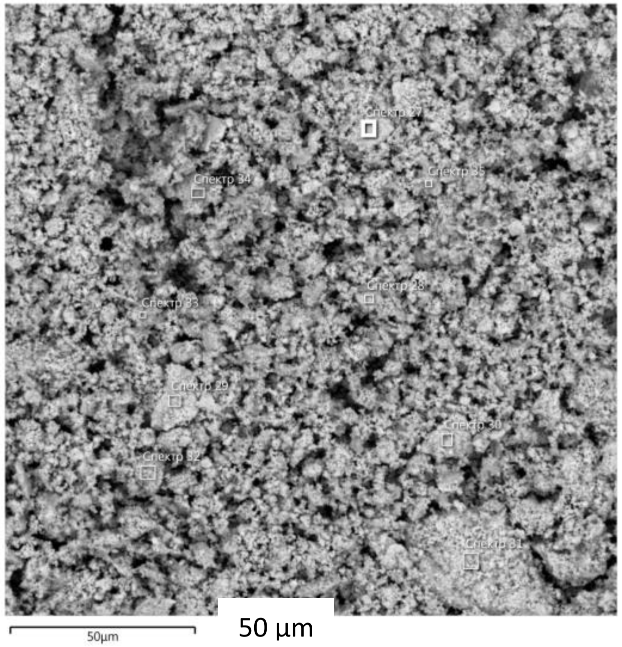
$Pb_{0.58}Eu_{0.42}F_{2.42}$	Pb<br>0.57±0.01<br><br>Eu<br>0.43±0.02<br><br>F<br>2.4±0.13	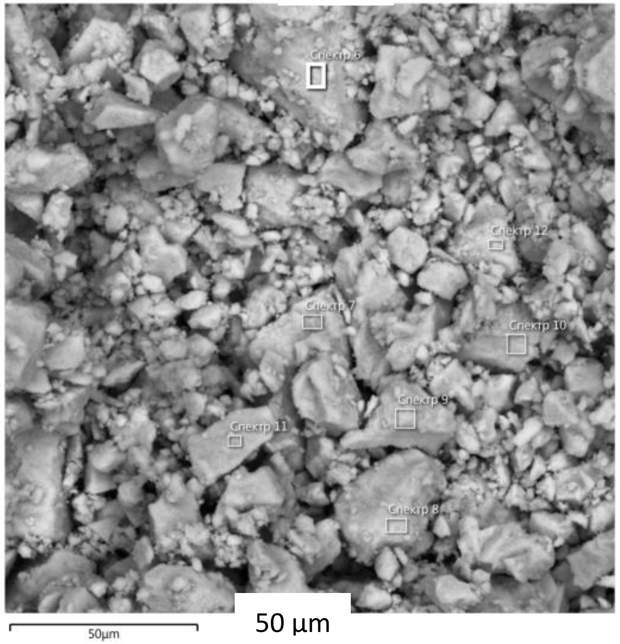		

$Pb_{0.55}Eu_{0.45}F_{2.45}$	Pb<br>0.55±0.01<br><br>Eu<br>0.44±0.02<br><br>F<br>2.47±0.14	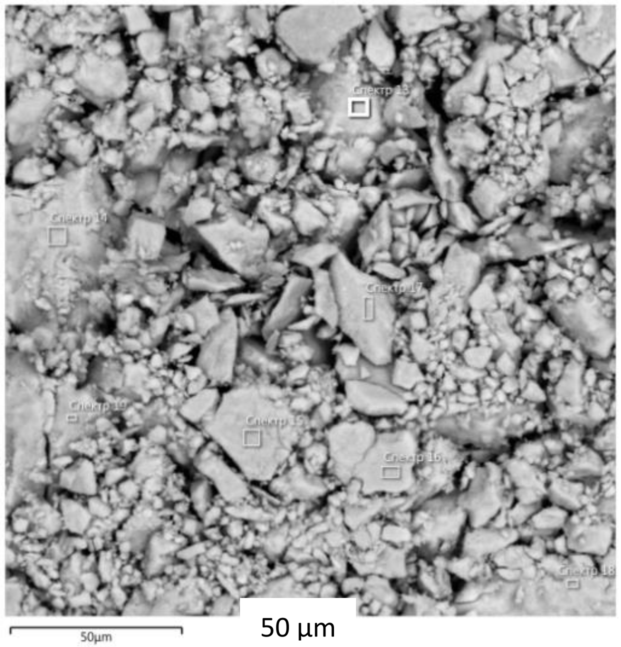		

Table S5. Cell parameter of $Pb_{1-x}Eu_xF_{2+x}$ samples (x=35-45 mol%)

Composition	Co-precipitation	Solid-phase synthesis	Calculated by Vegard law
	a, Å	*a*, Å	*a*, Å
$Pb_{0.65}Eu_{0.35}F_{2.35}$	5.85705	5.8233	5.8587
$Pb_{0.64}Eu_{0.36}F_{2.36}$	5.85468	5.8325	5.854
$Pb_{0.63}Eu_{0.37}F_{2.37}$	5.85231	5.823	5.853
$Pb_{0.62}Eu_{0.38}F_{2.38}$	5.84994	5.814	5.846
$Pb_{0.61}Eu_{0.39}F_{2.39}$	5.84757	5.813	5.8446
$Pb_{0.60}Eu_{0.40}F_{2.40}$	5.8452	5.8501	5.833
$Pb_{0.59}Eu_{0.41}F_{2.41}$	5.84283	5.8498	5.82374
$Pb_{0.58}Eu_{0.42}F_{2.42}$	5.84046	5.85	5.80382
$Pb_{0.57}Eu_{0.43}F_{2.43}$	5.83809	5.823	5.8211
$Pb_{0.56}Eu_{0.44}F_{2.44}$	5.83572	5.8477	5.8237
$Pb_{0.55}Eu_{0.45}F_{2.45}$	5.83335	5.8439	5.8327

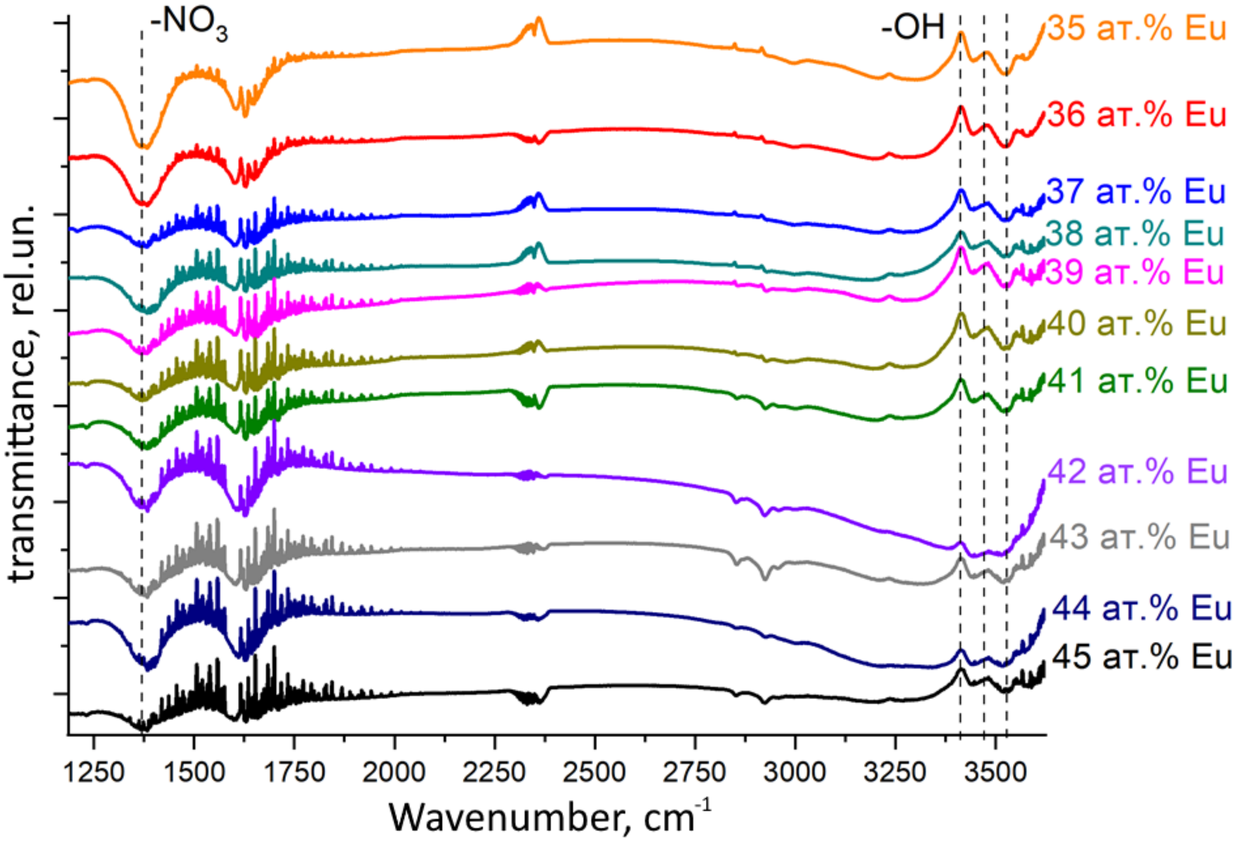

Figure S2. Fragment of the IR transmittance spectra of samples in the system (100-*x*)PbF_2-*x*EuF_3 (x=35-45 mol.% EuF_3), obtained by co-precipitation technique.

Table S6. Absorption band values for (100-*x*)PbF_2-*x*EuF_3 samples (x=35-45 mol.% EuF_3).

Co-precipitation technique, cm^{-1}	Solid phase synthesis, cm^{-1}	Notes
1370	-	-NO_3<br><br>Degenerate antisymmetric changes in bond lengths
3412	-	-OH<br><br>Valence vibration bands
3478	-	
3552	-	

Table S7. Raman spectra of solid solutions with different Eu content.

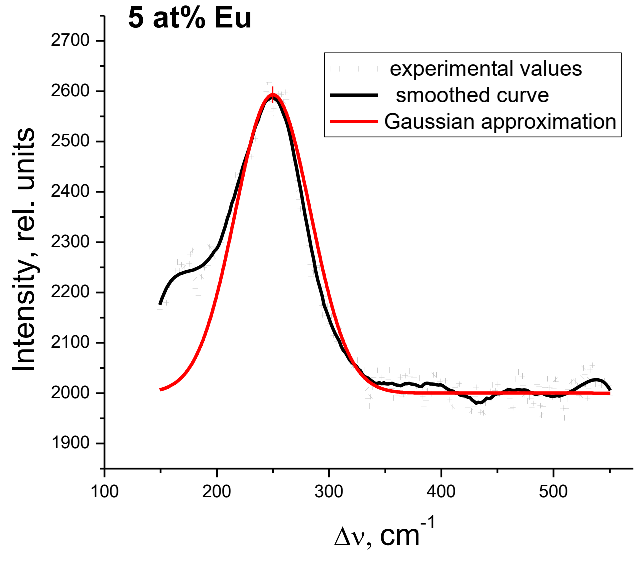

Parameters

		Value	Standard Error
Smoothed Y3	y0	2000	0
	xc	250	0
	w	67	0
	A	49789.03936	1014.94069
	sigma	33.5	0
	FWHM	78.88647	0
	Height	592.92397	12.08665

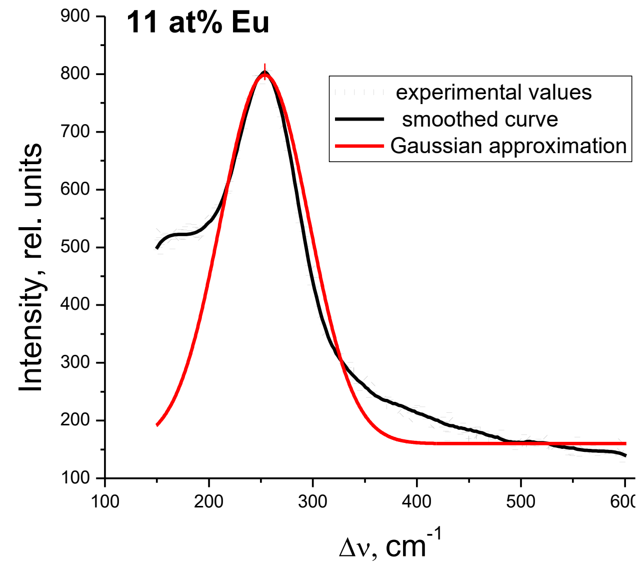

Parameters

		Value	Standard Error
Smoothed Y1	y0	160	0
	xc	253.73	0
	w	85	0
	A	68000	1519.27277
	sigma	42.5	0
	FWHM	100.07985	0
	Height	638.30765	0

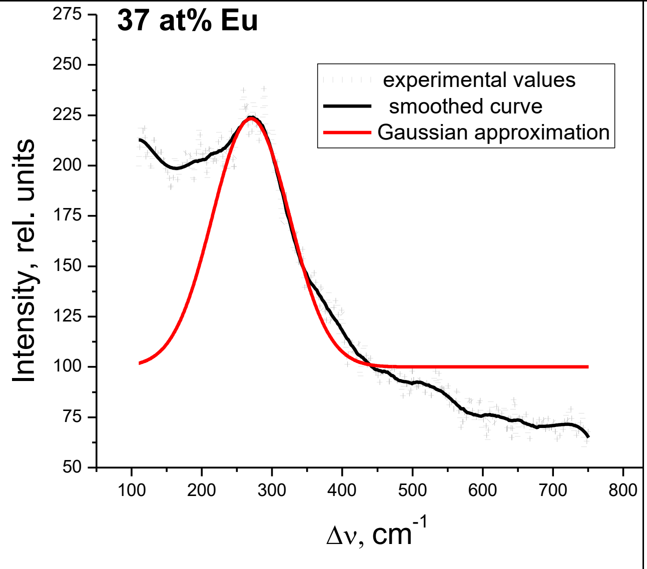

Parameters

		Value	Standard Error
Smoothed Y4	y0	100	0
	xc	270	0
	w	110	0
	A	17000	687.05488
	sigma	55	0
	FWHM	129.5151	0
	Height	123.30943	0

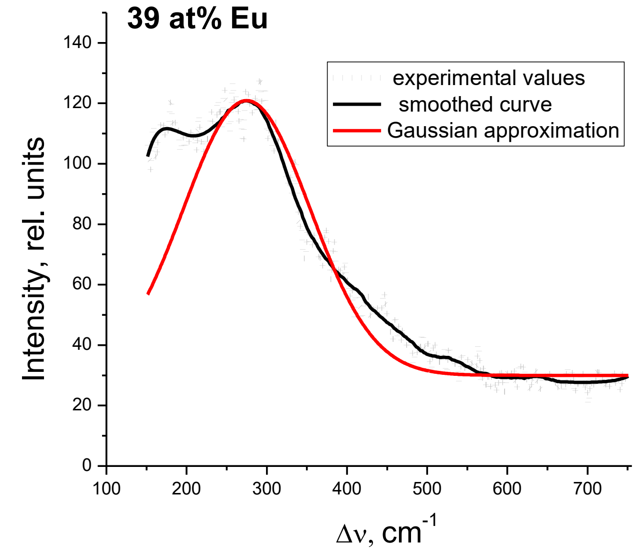

Parameters

		Value	Standard Error
Smoothed Y3	y0	30	0
	xc	275	0
	w	158	4.13595
	A	18000	388.50987
	sigma	79	0
	FWHM	186.03078	0
	Height	90.89824	0

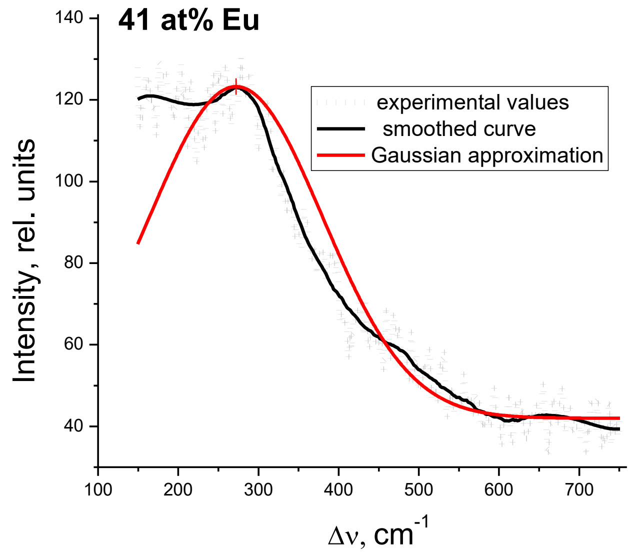

Parameters

		Value	Standard Error
Smoothed Y3	y0	42	0
	xc	272	0
	w	216	0
	A	22000	253.29469
	sigma	108	0
	FWHM	254.32056	0
	Height	81.26602	0

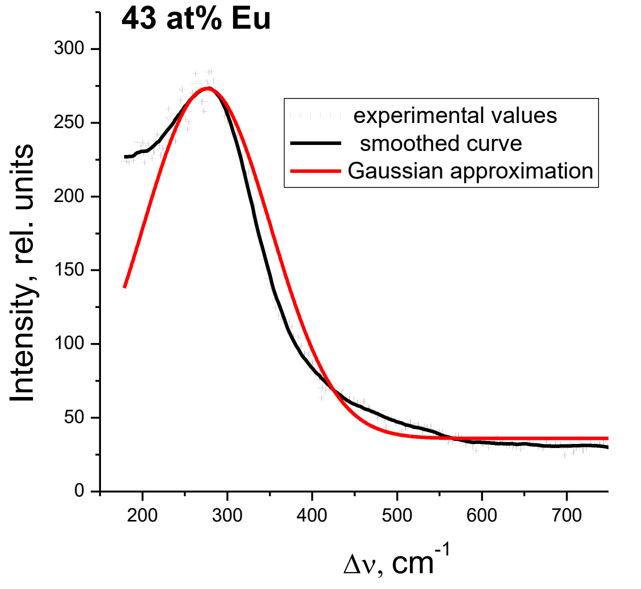

Parameters

		Value	Standard Error
Smoothed Y1	y0	36.0227	1.41651
	xc	276	0
	w	150	0
	A	44581.46555	572.57663
	sigma	75	0
	FWHM	176.6115	0
	Height	237.13909	0

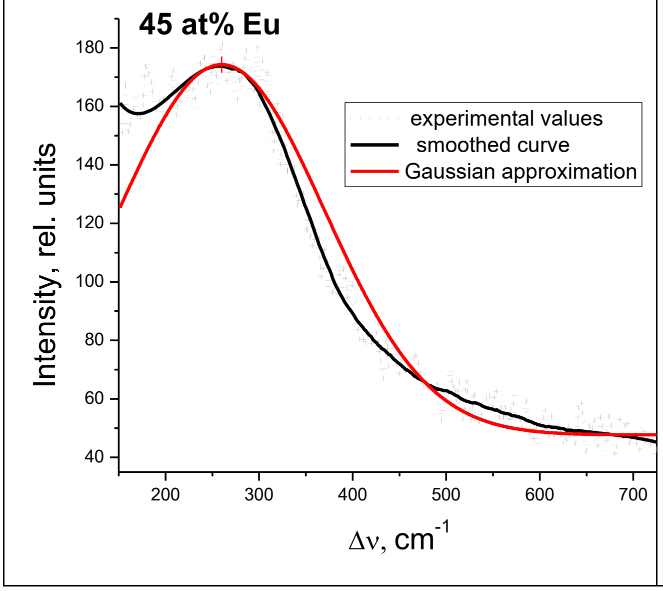

Parameters

		Value	Standard Error
Smoothed Y2	y0	47.6662	0
	xc	260	0
	w	220	1.75191
	A	34913.34434	0
	sigma	110	0
	FWHM	259.0302	0
	Height	126.6219	0

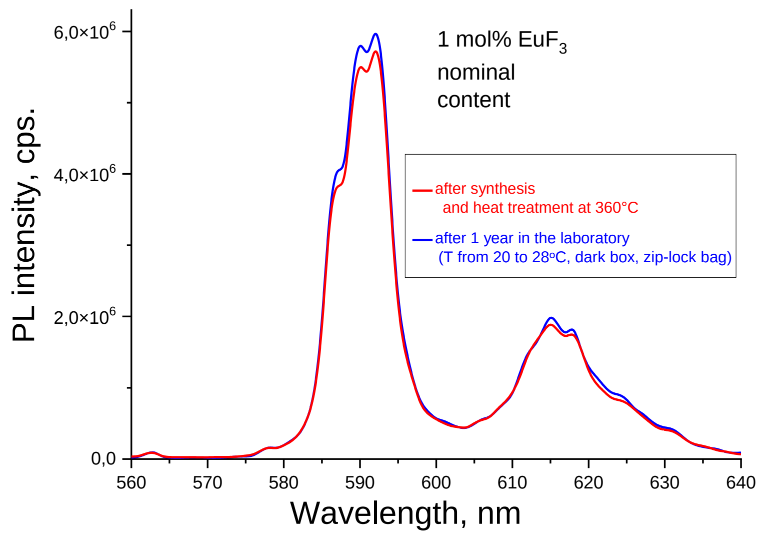
1 mol% EuF3
nominal
content
after synthesis
and heat treatment at 360°C
after 1 year in the laboratory
(T from 20 to 28ºC, dark box, zip-lock bag)
PL intensity, cps.
Wavelength, nm

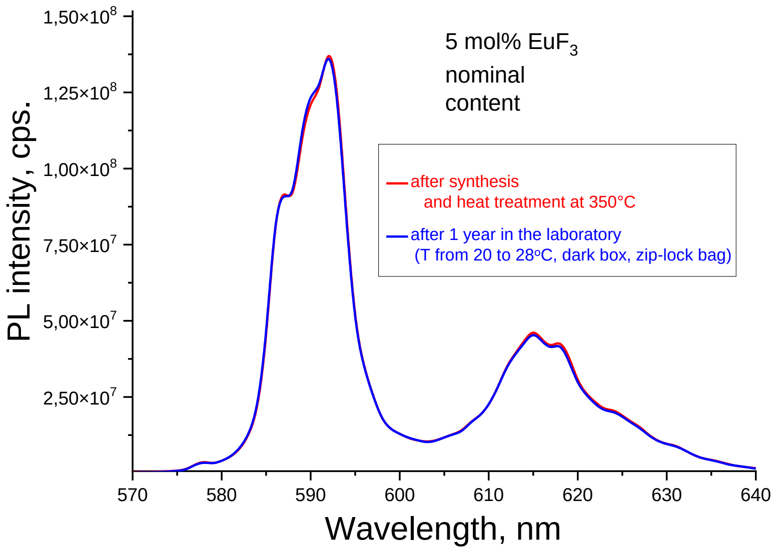
5 mol% EuF3
nominal
content
after synthesis
and heat treatment at 350°C
after 1 year in the laboratory
(T from 20 to 28ºC, dark box, zip-lock bag)
PL intensity, cps.
Wavelength, nm

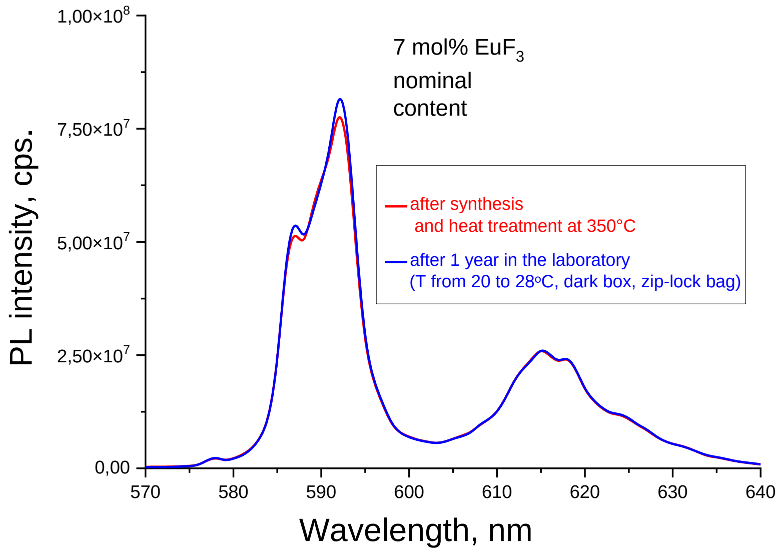

Figure S3. PL spectra of as-synthesized samples and after 1-year exposition under normal RT conditions.
